# A Scoping Review of Risk Factors of Postpartum Depression among Military Personnel and Spouses

**DOI:** 10.1093/milmed/usag005

**Published:** 2026-01-30

**Authors:** Catherine Biegel, Hailee Poulin, Sophia Silvia, Geoffrey McCullen

**Affiliations:** University of New England College of Osteopathic Medicine, Portland, ME 04103, United States; University of New England College of Osteopathic Medicine, Portland, ME 04103, United States; University of New England College of Osteopathic Medicine, Portland, ME 04103, United States; University of New England College of Osteopathic Medicine, Portland, ME 04103, United States

## Abstract

**Introduction:**

In recent years, there has been increased recognition and awareness surrounding mental health among US military personnel. However, the prevalence of postpartum depression (PPD) among female service members is significantly higher than that of the general US population. There is currently a gap in the literature characterizing the unique factors of the military experience accounting for the higher predisposition to PPD relative to civilian Americans. This qualitative review aims to address this gap by isolating risk factors which have been consistently identified in the available literature as contributing to the prevalence discrepancy.

**Materials and Methods:**

A MeSH database-built search utilizing key terms “postpartum depression” and “military” was used to gather sources that fit the inclusion criteria. These sources were analyzed for explicitly identified PPD risk factors pertaining to US military personnel and spouses.

**Results:**

Of the 14 sources meeting inclusion criteria, a *history of a mental health disorder* and a *lack of social support* were identified in seven distinct articles as significant risk factors for PPD among military personnel. *Low rank and pay*, as well as *deployments of self and/or spouse*, were specified in six papers. A *history of depression*, *history of anxiety*, and *exposure to combat* were identified in five papers. Four papers isolated *branch of service* and *young age* as risk factors. Factors that were recognized in three papers or less included, but are not limited to: *tobacco use*, *history of PTSD*, *history of sexual assault*, *low education attainment*, *high number of child dependents*, *race*, and *job stress*.

**Conclusions:**

Women in the military and spouses of military personnel are subject to an environment with distinct stressors, increasing their predisposition for PPD. Identifying specific risk factors is critical for quality screening, diagnosis, and care of PPD among this population. Significant risk factors consistently isolated from the available literature include history of mental health disorders, lack of adequate social support, low rank and pay, deployment of self or spouse, history of combat exposure, branch of service, and young maternal age. Findings of this review also suggest that early detection via thorough screening and disease mitigation by means of a 12-week maternity leave led to lower rates of PPD and better disease outcomes. Military-specific family health resources are widely available across branches of service and individual bases, though the use and quality of these resources are inconsistent. Providers of women experiencing at least one of these stressors should increase PPD precautions and refer patients to the appropriate preventative and acute care.

## INTRODUCTION

Included under a broader diagnosis of “peripartum depression” in the DSM-5, postpartum depression (PPD) is defined as a psychiatric mood disorder that typically presents within a few weeks after an individual has given birth. Symptoms can last up to a year and manifest as extreme or persistent sadness, anxiety, and fatigue. PPD is distinguished from the colloquial notion of “baby blues” by its more severe presentation, prolonged duration, and need for pharmaceutical treatment.^1^^,^^2^ PPD can affect a parent’s ability to bond with their newborn and the amount of time a mother chooses to breastfeed.^3^ In addition, the CDC reported in 2018 that 20% of maternal deaths in the first-year postpartum result from suicide. Although the role of PPD in these deaths is unclear, literature supports both that new mothers are vulnerable to PPD and that severe depression can include suicidal ideation. Thus, this condition should be treated with appropriate gravity.^4^

Robertson et al. conducted a systematic review to identify antenatal risk factors of PPD utilizing data from two meta-analyses and additional large-scale studies which evaluated in total about 24,000 subjects.^5^ Factor effects measured using Cohen’s *d* identified prenatal depression, prenatal anxiety, stressful life events during pregnancy or the early puerperium such as loss of a loved one or divorce, low levels of social support, and a previous history of depression as strong risk factors. This review also identified moderate risk factors including neuroticism and marital strain, as well as risk factors with small effect sizes including obstetric factors and socioeconomic status. These researchers recommended that healthcare providers apply clinical reasoning to identify women with these specific risk factors and follow them closely throughout the peripartum period.^5^ Treatment options for PPD among the general US population are well documented in the literature; first line interventions are efficacious and include selective-serotonin reuptake inhibitors and Cognitive Behavioral Therapy.^6^

In the United States, the prevalence of PPD among the general population is estimated at 13%, with variation by state.^7^^,^^8^ However, studies have found rates to be higher in a special subgroup: military personnel. A 2024 study by Wu et al. reported a PPD incidence of 15.9% using a sample of 4,178 active-duty soldiers in the Army who gave birth between January 2012 and December 2013.^9^ Another recent study by Pratt et al. with a sample size of 1,039 veterans representing various military branches demonstrated a prevalence of 26%, often with a concomitant diagnosis of postpartum anxiety or postpartum post-traumatic stress disorder (PTSD).^4^

As of June 2024, the Department of Defense (DoD) estimates that females constitute 17.9% of active-duty military personnel across all branches of service, including military academy cadets and midshipmen.^9^ Of female service members, 97% are within the reproductive age range, amounting to approximately 16,000 births by women on active duty each year.^10^^,^^11^ Of married servicewomen, 48% have active-duty partners; 7% of married servicemen have active-duty spouses. PPD risk factors are compounded in these families and extend to civilian women married to service men. Additionally, 12% of active-duty women are single parents, thus solely responsible for managing both childcare and self-care.^12^ Therefore, PPD poses a significant burden for women of United States military families.

Despite wide recognition of an elevated PPD prevalence among US military families compared to the general population, the breadth of research characterizing this discrepancy is limited. Service members and their spouses are known to endure distinct physical, emotional, and mental stressors, but it is unknown which specific aspects of the military experience predispose women to the development of PPD at higher rates; this review aims to address this deficit.

## METHODS

**Figure 1.**
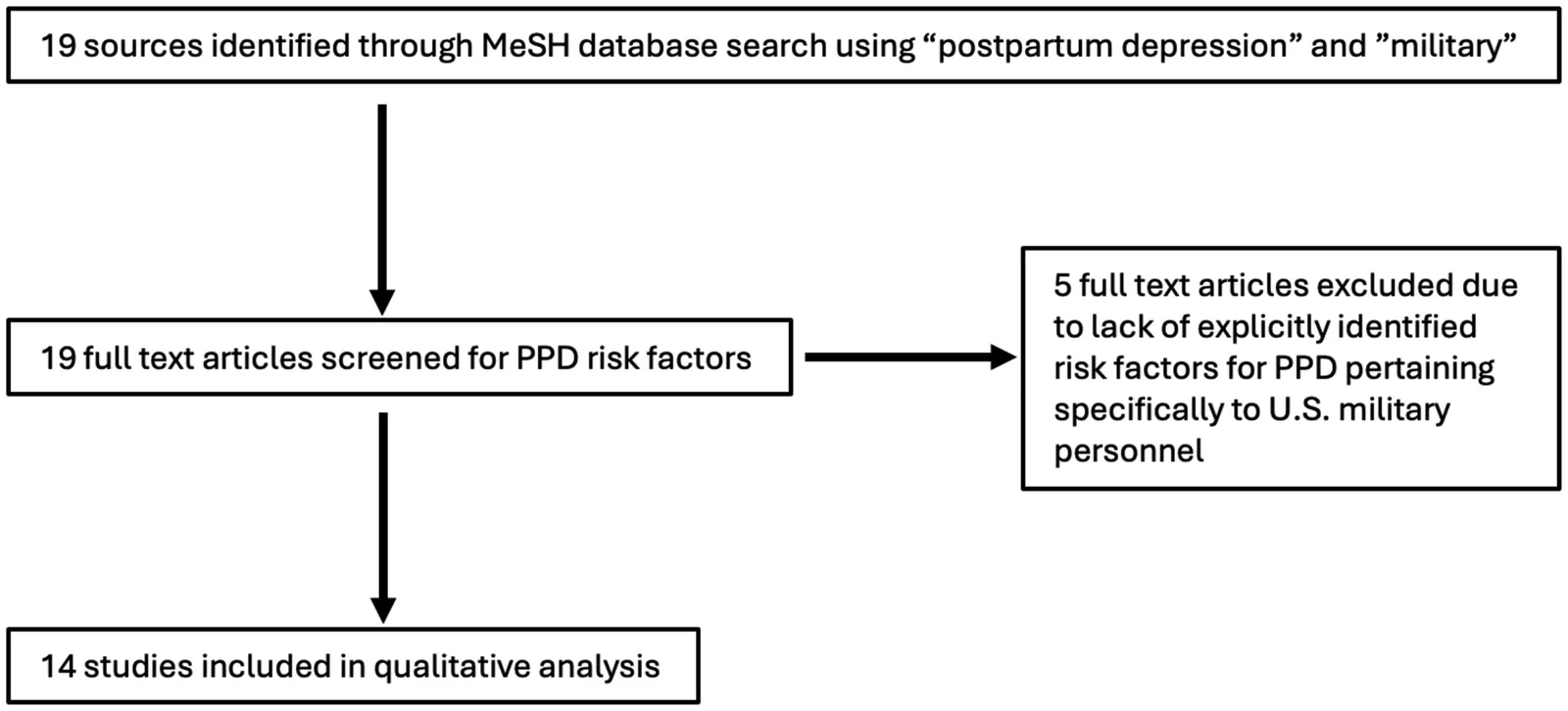
Schematic of the resource acquisition process via a MeSH heading database search and the inclusion criteria used to select publications in evaluation of military-unique risk factors for postpartum depression.

A MeSH database search using “PPD” and “military” yielded 19 sources from the following databases: AccessMedicine, ClinicalKey, JSTOR, PsycINFO, PubMed, and ScienceDirect. Fourteen papers met inclusion criteria which required the explicit identification or focused discussion of PPD risk factors relevant to American military personnel (Figure 1). The criteria were kept broad because of the limited pool of literature. Of the 14 papers, five were retrospective cohort studies, one of which was longitudinal; four were literature reviews; two were cross-sectional; one was mixed-methods; one was a prospective longitudinal cohort study; one was a quality improvement project with a prospective cohort design. PPD risk factors of interest were largely informed by existing literature, with three studies focusing specifically on factors related to deployment. Risk factors were assessed in these studies primarily through review of medical records and self-report via questionnaire or interview. In compilation, these sources reflected data on active-duty military members, reservists, veterans, and spouses across all military branches. Each article was analyzed for military-specific PPD risk factors and trends. The frequency with which each individual risk factor was isolated across the literature was quantified and compared. Risk factors were also categorized into three general classifications: past medical history, maternal social history, and family military status.

## RESULTS

**Figure 2.**
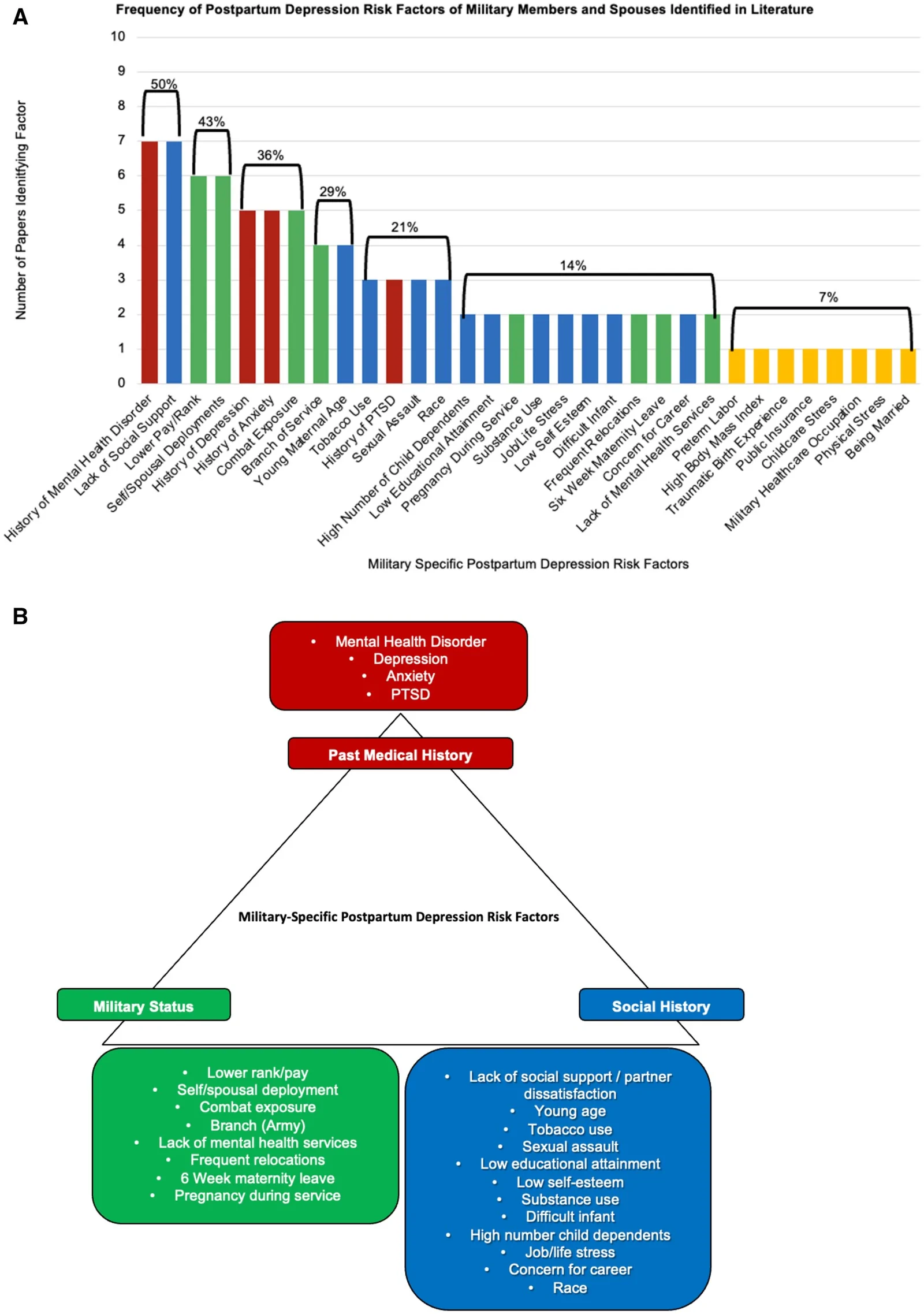
(A) Stratification of postpartum depression risk factors, specific to military personnel and spouses, based on the frequency with which they were identified in the literature. (B) Categorization of risk factors based on their contribution to an individual’s social, military, or medical experience. Factors that were identified only in one paper are not reflected and include: pre-term labor, high BMI, traumatic birth experience, public insurance, military healthcare occupation, physical stress, and being married.

Fifty percent of papers identified a *past medical history of mental health disorder* and a *lack of social support* as risk factors for PPD among military personnel (Figure 2).

Forty-three percent of papers specified *low rank* and *deployment of self or spouse* as significant risk factors. Broadly, higher rates of PPD were consistently reported among the enlisted, as opposed to officer class.^12^^,^^13^^,^^14^^,^^15^ Wu et al. demonstrated a distinction in PPD prevalence between the E5-E9 group and the E1-E4 reference group with an odds ratio of 0.64 (CI 0.49, 0.84, *P* < .01). The odds ratio between the W1-W5/O1-O10 group and the reference group was lower at 0.54 (CI 0.33, 0.89, *P* < .05).^9^

Wu et al. also reported that a higher number of maternal deployments before birth was a statistically significant predictor of PPD.^9^ In terms of spousal deployments, Spooner et al. found a higher prevalence of positive PPD screens among patients 6 weeks postpartum at Naval Hospital Camp Pendleton whose spouses were currently deployed, preparing to deploy, or recently returned from deployment relative to patients with no recent, current, or upcoming spousal deployments.^16^ The only patient group with significantly increased depression rates at the initial obstetrics encounter were those whose husbands had been currently deployed.^16^ Assessment of PPD among military spouses with partners deploying in the perinatal period by Smith et al. found that the risk for a positive depression screen was twice as likely compared to a non-deployed spouse control group.^17^

Thirty-six percent of papers cited a *history of depression, history of anxiety*, and *combat exposure* as risk factors. A history of depression and anxiety were both separately identified as among the strongest risk factors, in addition to the more general risk factor of past mental health disorders. Nguyen et al. found a prior depression diagnosis to have a 3.99 odds ratio compared to those with no depression history before PPD diagnosis (CI, 2.84-5.59, *P* < .01).^14^ Similarly, Wu et al. reported that women who had depression in the past had 2.75 times the risk of PPD relative to women with no depressive history; women who experienced depression during pregnancy had odds 7.91 times that of women with no history.^9^ Regarding combat, Nguyen et al. found exposure specifically to combat to be a greater predictor of depression than general deployment.^14^

Twenty-nine percent of papers recognized *branch of service* and *young maternal age*. Nicholson et al. found that there were higher incidence rates among the Army and Air Force than among the Navy and Marine Corps.^12^ Identification of the Army as the branch with the highest rates of PPD was present across multiple studies.^13^^,^^18^ Herrick et al. found active-duty women with PPD to be overall younger than women without PPD, with the average age of these groups being 26.3 and 27.1, respectively (*P* < .01).^13^ Nguyen et al. stratified participants by those born before 1970, between 1971-1979, and after 1980. (These cohorts were first surveyed for childbirth from 2004 to 2006, making the latter group 26 years old or younger.) With the pre-1970 group serving as the reference group, the 1980-present group had an odds ratio of 2.13 (CI 1.27, 3.57, *P* < .01).^14^ Nicholson et al. found that most participants with PPD were in the 20-24 age group, with other groups being under 20 years, 25-29, 30-34, 35-39, and 40 or older.^12^

*Tobacco use*, *sexual assault*, *history of PTSD*, and *race* were risk factors identified by three papers. Pratt et al. found that 56.2% of 1,039 qualifying survey respondents (veteran women) experienced a completed or attempted sexual assault, with 570 women having been sexually assaulted before their most recent pregnancy.^4^ In this study, history of sexual assault had odds ratios of 1.8 (CI 1.3, 2.4) and 2.9 (CI 1.8, 4.7) for PPD and postpartum PTSD, respectfully. Associations in which the confidence interval did not include 1 were considered statistically significant by the study.^4^ Nyugen et al. reported a 6.76 odds ratio for PPD associated with a prior PTSD diagnosis (CI 4.50, 10.15, *P* < .01).^14^

Regarding race, Nicholson et al. found a higher rate of PPD among White individuals relative to Black and “Other Category” subpopulations.^12^ Wu et al. reported no significant difference in PPD prevalence among Black and White subgroups but a decreased odds ratio of 0.63 for Asian and Pacific Islander relative to the White reference group (CI 0.41, 0.97, *P* < .05).^9^ Herrick et al. identified the majority of PPD patients as “non-White.”^13^

Two papers specified a higher *number of child dependents, low educational attainment*, *pregnancy during service, substance use, stress related to career* or *life circumstance, low self-esteem, difficult infant, frequent relocations, 6-week maternity leave, concern for career,* and *lack of mental health services*. Wu et al. identified an odds ratio of 1.32 in mothers with two or more child dependents compared with no child dependents (CI 1.01, 1.72, *P* < .05). This study also found that among soldiers with PPD, 68.7% obtained a high school diploma or equivalent, 16% attended some college, 10.7% obtained a Bachelor’s degree, and 4.7% obtained a graduate degree (*P* < .001); however, odds ratio comparisons between education levels with high school education as the reference group were not statistically significant.^9^ Nguyen et al. reported a protective odds ratio of 0.43 for participants who had attained a Bachelor’s degree or higher relative to some college or less (CI 0.24, 0.79, *P* < .01).^14^ Meanwhile, the following factors were only reported in one of the fourteen papers and are included here for comprehensive purposes: *preterm labor, high BMI, traumatic birth experience, public insurance, childcare stress, military healthcare organization, physical stress*, and *being married*.

## CONCLUSION

Categorization of variables allowed for the visualization of mental health and military status factors as the most frequently identified PPD risk factors among military personnel and spouses (Figure 2). Specifically, analysis of the literature isolated a past medical history of a mental health disorder, lack of social support, low rank (a proxy for socioeconomic status), deployments of self-and/or spouse, history of depression, history of anxiety, and combat exposure as the most prevalent PPD risk factors. Lack of social support was referred to in multiple papers as a comprehensive description for circumstances to which deployment, lack of mental health services, frequent relocations, single motherhood, limited maternity leave, and other factors may contribute. Thus, the authors’ mention of “social support” ultimately describes both the social and military experience of new mothers.

Compromised mental health before and during pregnancy, in combination with inadequate social support systems, corresponds to the extensively documented predisposing factors for PPD reported in civilian cohorts.^5^ Yet, discrepancy in reported PPD prevalences between the general and military population exists despite this overlap in contributory factors. A plausible explanation is higher rates of mental health disorders among military members compared to the general US population, as well as unique social challenges faced by military families. The Health Related Behaviors Survey of 2015 conducted by the US DoD and the RAND Corporation displays higher rates of anxiety, depression, and PTSD in active-duty military personnel [including men] in comparison to the civilian population that year.^19^ The military experience entails physical and psychological traumas not faced by civilians; furthermore, they encounter increased barriers to what is conventionally considered a “stable” lifestyle.^19^

In addition, socioeconomic status was listed as a less impactful risk factor in Robertson et al., although playing a relatively prominent role with consistent associations among military samples. Although consistently significant, more research is needed to analyze the strength of the association between low socioeconomic status and PPD among military populations, as well as the interplay between socioeconomic status and other military hardships which may compound one another.

The general impact of deployment has been a more widely researched topic, with diffuse agreement of its toll on mental health outside the literature included in this review. A 2010 study by Danielson et al. found that women who deployed 24 months postpartum had a 15% lower rate of mental health disorders compared to women deploying between 12 and 24 months after delivery. Those deploying within 6 months had a 37% higher rate compared to the 12-to-24-month group.^20^ In addition, Nguyen et al. noted that members of the Army tend to deploy more often and for longer duration than other branches,^14^ which could indicate that deployment is a confounding variable for branch of service.

Discussions regarding race among the included literature were inconsistent, despite agreement that race itself is a significant PPD risk factor. Further research is needed to analyze larger sample sizes across multiple populations for a better understanding of the role of racial identification in PPD. The protective effect demonstrated by Asian and Pacific Islander participants in Wu et al. offers insight that race has the potential to be both a risk and a protective factor.^9^

It should also be considered that risk factor prevalence may misrepresent some factors which are strongly associated with PPD but were included in fewer studies. For example, sexual assault is a traumatic event known to increase the risk of depression and PTSD. If this factor had been explicitly assessed in a greater proportion of studies, it might have been identified as a more prevalent risk factor. These relationships are especially significant considering the cyclical trajectory of mental health disorders, which frequently confer heightened vulnerability to future psychological morbidity. Further research is needed to determine the strength of association of risk factors identified in this study.

Although the factors isolated in this review are somewhat expected by the healthcare community, it is important to identify specific factors contributing to the disease prevalence discrepancy between civilian and military personnel to better inform efforts for prevention and detection. The studies of this review consistently acknowledge [lack of] social support and mental health history. Therefore, mental healthcare should be universally promoted among service members, with heightened attention to those with particular experiences, to ensure that mental health conditions are optimally managed before pregnancy. For example, Nguyen et al. emphasized the impact of combat exposure on mental health, which may indicate a need for bolstered reintegration programs and increased mental health screenings post combat-associated deployment.^14^ Pratt et al. speaks to the importance of clinician awareness of patient sexual assault history so that trauma-informed care may be provided, wellness optimized, and military readiness reinforced. Thus, easily accessible mental health resources, and standardization of mental healthcare professional training to facilitate the use of those resources, may mitigate the perpetuation of mental distress postpartum.^4^

In addition, it is reasonable that all pregnant service members or spouses of military members receive a formal orientation to pregnancy and lactation resources, as well as introductions to support groups and childcare services, during their prenatal care. Familiarity with these resources and how to access them before childbirth may make their utilization more likely in the postpartum period when the mother may be physically and emotionally overwhelmed. The idea that engagement with community support systems is protective is endorsed by Spooner et al., which was a study conducted with data from Marine Corps Base Camp Pendleton.^16^ Naval Hospital Camp Pendleton sponsors semi-monthly on-base meetings of a group called MIST, Maternal Infant Support Team. At these meetings, families have a chance to interact with social workers, healthcare providers, community program representatives, and other support organizations. Interestingly, Spooner et al. found lower than expected rates of PPD at 4.6% despite similar demographic distributions to other US military populations which were reporting rates higher than 20%.^16^ Although all bases have established resources available to military personnel including, but not limited to, routine healthcare appointments, birthing classes, and lactation counseling at no-cost through TriCare, reports vary regarding the use and efficacy of these services specifically relative to PPD.^15^ Therefore, models similar to MIST where families can engage with resources in meaningful and proactive ways may better serve perinatal mothers.

Postpartum care should emphasize quality bonding between mother and baby. Herrick et al. reported a 50% decrease in the odds of PPD from 2016 to 2017, following a 2016 policy change which extended maternity leave to 12 weeks across branches, compared with 2011 to 2015 numbers reflecting a maternity leave of 6 weeks.^13^ Although this study is not alone in suggesting that longer maternity leave is protective against PPD, the 12-week timeline is somewhat arbitrary. The Maternity Protection Recommendation set forth by the International Labor Organization in 2000 recommends a minimum of 18 weeks maternity leave, with extensions for multiple gestation pregnancies.^21^ Thus, more research is needed to determine the true maternity leave duration safest for mother and baby, which may be beyond 12 weeks. However, the 2016 mandate provides optimism that awareness of PPD among the military community is growing, and efforts are being put toward prevention and intervention. Not only do these policy guidelines and resources need to exist, but patients should be empowered to seek the protected maternity benefits to which they are entitled.

When PPD prevention proves insufficient, the key to recovery is early detection and treatment. Current screening tools have shown to be effective in identifying PPD, despite the lack of standardization. These tools include the Edinburgh Postnatal Depression Scale (EPDS), the Beck Depression Inventory, the Postpartum Depression Predictors Inventory-Revised, and the Postpartum Depression Screening Scale.^6^^,^^22^ The EPDS was used in 70% of the studies reviewed. Herrick et al. found that rates of PPD across branches increased by more than 3% in 2018 relative to 2017 and rose almost another 4% in 2019.^13^ This study postulated that the increase in PPD diagnoses was a result of the 2018 Veteran Affairs and DoD Clinical Practice Guideline on Pregnancy Management which reinforced the importance of depression screening during pregnancy and postpartum.^13^

A study on the pilot program, *HealthySteps*, by Chooey et al. offers another approach to PPD identification.^23^*Healthy­Steps* was established in January of 2018 to promote pediatric care and provide resources for military families as frequent relocations, long work hours, and parent deployments create unique hardships. This study demonstrated that participating families were not only screened more frequently for PPD compared to the control group but were more likely to talk about their symptoms.^23^ In addition, program participants received a higher number of community resource referrals.^23^ Such program models therefore have the potential to be both protective against PPD and helpful in identifying patients needing anti-depressive treatment.

This review adds to current literature by isolating specific risk factors of the military experience which call for heightened PPD precautions by providers, as well as strategies to mitigate PPD as a public health issue faced by the military. More research is necessary to evaluate the efficacy of current preventative, interventional mental health, and new parent resources—as well as factors which may deter their utilization such as fear of career repercussions.^24^ The promotion of available professional and community support, diligent PPD screening, maternity leave, and the encouragement of patients will aid in the protection of our service members who experience childbirth.

## Data Availability

No new data was generated or analyzed in support of this research.
